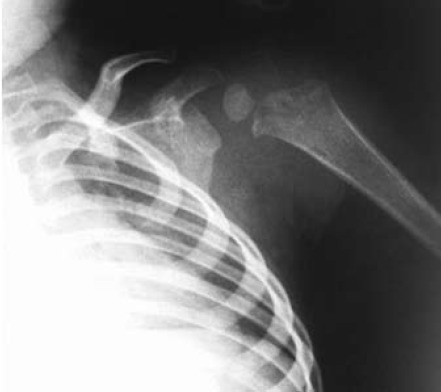# Salmonella Osteomyelitis in a one year old Child without Sickle Cell Disease: A Case Report

**DOI:** 10.5704/MOJ.1407.005

**Published:** 2014-07

**Authors:** C Saturveithan, A Arieff, G Premganesh, N Sivapathasundaram

**Affiliations:** Depertment of Orthopaedics, Malacca Hospital, Malacca, Malaysia; Depertment of Orthopaedics, Malacca Hospital, Malacca, Malaysia; Depertment of Orthopaedics, Malacca Hospital, Malacca, Malaysia; Depertment of Orthopaedics, Malacca Hospital, Malacca, Malaysia

## Abstract

A one year old boy was admitted with left shoulder pain with reduced range of motion of five days’ duration associated. Inflammatory markers were raised and radiograph of the left shoulder revealed widening of the metaphysis of the proximal humerus. Empirically, he was started on intravenous C-penicillin and cloxacillin after initial joint aspiration yielded only synovial fluid with negative culture. Subsequent MRI revealed acute osteomyelitis of the proximal left humerus with concurrent septic arthritis of the shoulder joint. Cultures from the arthrotomy washout grew Salmonella sp, sensitive to ampicillin. He recovered following six weeks of intravenous unasyn ( ampicillin and sulbactum). This rare case of salmonella osteomyelitis in a non-sickle cell disease patient was diagnosed with serial laboratory and radiological studies and was successfully treated with adequate duration of antibiotics and operative intervention.

**Key Words:**

salmonella osteomyelitis, non-sickle cell disease, children

## Introduction

In general, Salmonella sp. is an unusual causative agent of osteomyelitis and is exceedingly rare in non-sickle cell disease patients. It often presents with atypical clinical and radiographic findings. We report an unusual case of salmonella osteomyelitis in a child without sickle cell disease.

## CASE REPORT

A one year old boy had been admitted for acute pharyngitis. His admission was uneventful and he was subsequently discharged well after two days. He was re-admitted one week later with left shoulder pain and reluctance to move the limb. According to his mother, he had developed the symptoms three days after being discharged from hospital. She denied any trauma or any illness besides for the recent pharyngitis.

On examination he was afebrile and appeared generally well. His left shoulder was not swollen or tender but there was significant reduction in active range of motion at the joint. A full blood count revealed haemoglobin of 9.2g/ dl, total white cell at 11.3g/L and platelets at 576g/L. Erythrocyte sedimentation rate (ESR) and C-Reactive protein (CRP) were 109mm/hr and 47.4mg/L respectively. Radiographs revealed widening of the metaphysis of proximal humerus. Joint ultrasonography displayed anterior soft tissue swelling with thickened synovium with a collection with a fluid collection measuring 1.0 x 0.9 x 1.7 centimetres. The proximal humerus showed a hypoechoic area with muscle septations. In keeping with the findings he was diagnosed with septic arthritis and underwent an arthrotomy washout. Intraoperatively, there was no pus but only five cubic centimetres of synovial fluid which was sent for cytology, culture and sensitivity, and acid fast bacilli (AFB) culture and sensitivity.

As intraoperative findings were not suggestive of septic arthritis, a differential diagnosis of proximal humerus osteomyelitis was made. Empirically, intravenous penicillin and cloxacilin were started and the left upper limb was immobilized in an arm sling. ESR and CRP were taken weekly to monitor treatment response. Postoperative follow-up showed an improved shoulder motion. Synovial fluid cultures did not yield any growth. CRP after a week showed an increase to 57.4mg/L. The ESR remained unchanged. Plain radiographs at two weeks revealed acute osteomyelitis changes with lytic lesions in the proximal humerus. Blood culture and sensitivity, urine and stool culture and sensitivity did not yield any growth. Synovial fluid cytology taken was in line with acute inflammatory response.

MRI of the left shoulder was done eleven days after admission in view of the persistent shoulder pain and static inflammatory markers. It revealed acute osteomyelitis of the proximal metaphysis and diaphysis of the left humerus with marrow involvement. Shoulder joint septic arthritis was suspected due to a large joint fluid collection, multiple intramuscular abscesses and axillary lymphadenitis. He underwent a second arthrotomy washout and debridement of the left shoulder joint. Pus and slough from the joint and surrounding tissue which were sent separately for culture and sensitivity grew Salmonella sp, sensitive to ampicillin. Subsequently, antibiotics were changed to intravenous unasyn ampicilin and sulbactam. He responded well as evidenced by clinical and radiological improvement. The sickle cell disease workup which included Haemoglobin electrophoresis was negative. Throat swab culture were subsequently reported to be negative.

Three weeks following admission he was discharged well with a markedly improved shoulder motion. Oral antibiotics were continued for six weeks of duration. Follow-ups were done three weekly with radiographs and ESR/CRP on each follow-up, and then extended to six weekly intervals. The ESR normalized within three months. He had a full range of motion of the shoulder when reviewed after a year, with normal inflammatory markers. The radiographs showed bone healing without significant physeal damage.

## Discussion

Salmonella infections may manifest in five different clinical forms namely, gastroenteritis, enteric fever, bacteraemia focal disease (including soft tissue infection), and the chronic carrier state ^1^. Salmonella osteomyelitis is very rare in normal children amounting to 0.45% of all osteomyelitis ^1^. About 0.8% of cases of typhoid fever developed Salmonella osteomyelitis ^1^. The three most common strains of salmonella causing osteomyelitis are Salmonella typhimurium, Salmonella typhi, and Salmonella enteritidis, with Salmonella typhi being the only strain to be transmitted from human to human ^1,2^. The infection is associated with sickle-cell disease, hemoglobinopathies, malignancies, and liver disease. Most common mode of spread is hematogenous and the most frequent sites involved are the diaphysis of long bones mainly the femur and humerus ^2^. Twenty-one cases of salmonella osteomyelitis in healthy children has been reported in the United States from 1978 till 2012, of whom, 45% had involvement of the long bones, mainly the diaphysis followed by pelvic bones and vertebrae ^3^.

Diagnosis is often delayed as in the acute phase, as plain radiographs offer no aid as signs of osteomyelitis do not appear until 10 to 14 days after onset of symptoms ^4^. Certain radiographic features associated with salmonella osteomyelitis such as nonspecific osteolytic foci with sclerotic margins may mimic bony tumours ^4^.

MRI is often helpful in diagnosing osteomyelitis earlier compared to plain radiographs. With regards our case, MRI helped us to come to the diagnosis, whereupon subsequently debridement and curettage was done and the intraoperative cultures led us to the definitive diagnosis of Salmonella osteomyelitis. Although blood culture was negative for Salmonella sp in our case, positive results has been reported in only 71% of patients with Salmonella osteomyelitis ^5^.

The antibiotic treatment should ideally be initiated after obtaining sufﬁcient samples from bone for microbiological assay ^5^. Most of the studies stated that the most effective therapy of a confirmed salmonella osteomyelitis is a combination of radical operative intervention and targeted intravenous antibiotics as in our case ^5^.

Salmonella osteomyelitis clinically and radiologically indistinguishable from osteomyelitis caused by other organisms. Adequate duration of antibiotics sensitive to the microbiological assay in addition to operative intervention offer a good outcome.

Table I

Figure 1

Figure 2

Figure 3

**Table I: Serial laboratory investigations which were taken on Days 1, 7, 10, 15 and 21 of admission respectively.**
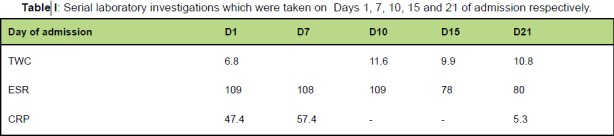


**Fig. 1: Anteroposterior radiograph of left shoulder showing widening of the joint space with lytic lesion of the metaphysis of proximal humerus.**
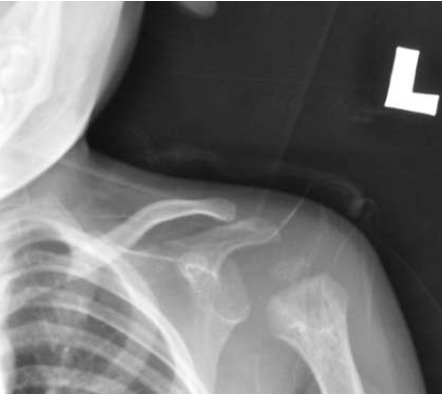


**Fig. 2: Radiograph of left shoulder at 2 weeks of admission demonstrates lytic and sclerotic changes of the left proximal humerus consistent with osteomyelitic changes.**
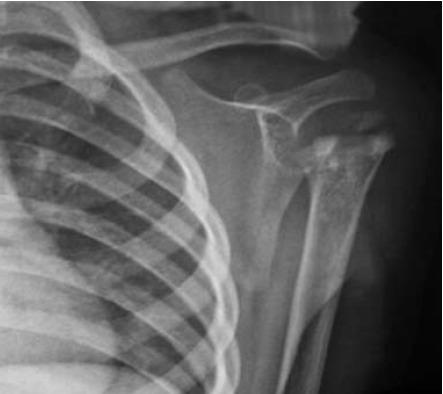


**Fig. 3: Radiograph of left shoulder at 6 weeks demonstrates marked improvement of the proximal humerus of the left shoulder in comparison with radiographs taken at 2 weeks.**